# Detecting Spurious Correlations With Sanity Tests for Artificial Intelligence Guided Radiology Systems

**DOI:** 10.3389/fdgth.2021.671015

**Published:** 2021-08-03

**Authors:** Usman Mahmood, Robik Shrestha, David D. B. Bates, Lorenzo Mannelli, Giuseppe Corrias, Yusuf Emre Erdi, Christopher Kanan

**Affiliations:** ^1^Department of Medical Physics, Memorial Sloan Kettering Cancer Center, New York, NY, United States; ^2^Chester F. Carlson Center for Imaging Science, Rochester Institute of Technology, Rochester, NY, United States; ^3^Department of Radiology, Memorial Sloan Kettering Cancer Center, New York, NY, United States; ^4^Institute of Research and Medical Care (IRCCS) SDN, Institute of Diagnostic and Nuclear Research, Naples, Italy; ^5^Department of Radiology, University of Cagliari, Cagliari, Italy

**Keywords:** deep learning, computed tomography, bias, validation, spurious correlations, artificial intelligence

## Abstract

Artificial intelligence (AI) has been successful at solving numerous problems in machine perception. In radiology, AI systems are rapidly evolving and show progress in guiding treatment decisions, diagnosing, localizing disease on medical images, and improving radiologists' efficiency. A critical component to deploying AI in radiology is to gain confidence in a developed system's efficacy and safety. The current gold standard approach is to conduct an analytical validation of performance on a generalization dataset from one or more institutions, followed by a clinical validation study of the system's efficacy during deployment. Clinical validation studies are time-consuming, and best practices dictate limited re-use of analytical validation data, so it is ideal to know ahead of time if a system is likely to fail analytical or clinical validation. In this paper, we describe a series of sanity tests to identify when a system performs well on development data for the wrong reasons. We illustrate the sanity tests' value by designing a deep learning system to classify pancreatic cancer seen in computed tomography scans.

## 1. Introduction

Artificially intelligent (AI) computer-aided diagnostic (CAD) systems have the potential to help radiologists on a multitude of tasks, ranging from tumor classification to improved image reconstruction (1–4). To deploy medical AI systems, it is essential to validate their performance correctly and to understand their weaknesses before being used on patients (5–8). For AI-based software as a medical device, the gold standard for analytical validation is to assess performance on previously unseen independent datasets (9–12), followed by a clinical validation study. Both steps pose challenges for medical AI. First, it is challenging to collect large cohorts of high-quality and diverse medical imaging data sets that are acquired in a consistent manner (13, 14). Second, both steps are time-consuming, and best practices dictate limited re-use of analytical validation data. The cost of failing the validation process could prohibit further development of particular applications.

One reason AI systems fail to generalize is that they learn to infer spurious correlations or covariates that can reliably form decision rules that perform well on standard benchmarks (15). For example, an AI system successfully trained to detect pneumonia from 2D Chest X-rays gathered from multiple institutions, but it failed to generalize when images from new hospitals outside of the training and assessment set were used to evaluate the system (16). The investigators found that the system had unexpectedly learned to identify metal tokens seen on the training and assessment images (16). In hindsight, the tokens were obvious spurious correlators, but in other cases, the covariates can be less obvious (15). For example, subtle image characteristics that may be unrelated to the target object, such as high-frequency patterns (17–19), object texture (20, 21), or intangible attributes of objects are known to cause AI systems to form decision rules that may not generalize (15, 22). Current research has focused on explaining or interpreting AI decisions using various visualization techniques (23), but these do not necessarily imply that a system will generalize (24–27).

Addressing system failures before clinical deployment is critical to ensure that medical AI applications are safe and effective. Identifying systems that are right for the wrong reasons during the development stages can expedite development by not wasting valuable validation data from multiple institutions or conducting doomed clinical validation studies.

The standard approach used to identify system failures involves testing with held-out development or generalization test datasets (28). However, development test sets are subsets of the training data, and their primary value lies in identifying systematic errors or bugs within the AI algorithm. Generalization test data are independent of the development data (i.e., their joint probability distribution of inputs and labels differ from training and development test data) (29). The generalization data's value is to assess how well a trained model may adapt to previously unseen data. However, neither type of test is sufficiently robust enough to declare when an AI system is ready for the clinic.

We provide a set of sanity tests that can demonstrate if a trained system is right for the wrong reasons. We developed a weakly supervised deep learning system for classifying pancreatic cancer from clinical computed tomography (CT) scans to illustrate their use. Our main contributions are:

1. We provide a set of sanity tests to determine if a system is making predictions using spurious correlations in the data.
2. We describe a system for using deep learning with CT images to detect pancreatic cancer, and we apply our set of sanity tests to both development and generalization test datasets. We train and assess four unique variants of this system to illustrate the pipeline and demonstrate that the system looks as if it performs well in many scenarios, but it is predicting using spurious correlations.
3. We illustrate how to use a method to generate noise images from the patients' volumetric CT scans. These can then be used to assess the influence of noise on the AI system's performance.

## 2. Materials and Methods

### 2.1. Sanity Tests for AI Systems

There are various testing procedures employed in software engineering to determine if a system is working correctly, such as smoke and sanity tests (30). Smoke tests evaluate the critical functionality of a system before conducting additional tests. In AI, this is analogous to reaching an acceptable level of performance on the development test data, which matches the training data's distribution. Development test data is typically a random sample of the training data (e.g., 30% test and 70% train). The stopping point for many AI projects is when acceptable performance is achieved on the development test set, but in software engineering, the next step is to conduct ‘sanity tests’ that indicate if a system produces obvious false results. If the sanity tests fail, further development is done before conducting more time-consuming and rigorous tests, which for AI systems used in medical applications could correspond to analytical and clinical validation studies. For AI systems, sanity tests would identify if a system is achieving good results on the development test set for the wrong reasons (e.g., covariates or spurious correlations) and will therefore fail in other environments or on other datasets.

Sanity tests are occasionally used to identify if a system is unlikely to generalize (24, 31, 32). However, the tests are often designed to evaluate literature methods instead of being used as a crucial development tool. For example, Shamir et al. critiques the methods by which face datasets were designed and evaluated by showing that commonly used face recognition datasets were classified correctly even when no face, hair, or clothing features appeared in the training and testing datasets (33). As another example, in response to a report suggesting AI systems could diagnose skin cancer at the level of dermatologists (34), Winkler et al. evaluated the limits of the claim by testing a trained AI system using dermoscopic images where the covariate's, hand-drawn skin markings, were first present and then absent from pictures of the skin cancer. They observed that when skin markings were present, the probability that the AI system classified images as having skin cancer increased significantly. With the markings removed, the probability decreased, which led them to conclude that the AI system associated the markings with cancer instead of the actual pathology (31).

For AI-based medical devices, conducting sanity tests can prevent needless harm to the patient and save a considerable resources. However, without sufficiently large, well-annotated datasets, performing analytical validation to determine the root causes that drive AI systems to fail before deployment remains a challenge (5, 35). Moreover, after independent testing data is gathered, regulatory organizations advise that the data be used a limited number of times to prevent over-fitting (36). For example, the United States Food and Drug Administration “discourages repeated use of test data in the evaluation” of CAD systems (37). Clinical validation of deployed systems is likewise time-consuming to organize and often costly.

**Figure 1:**
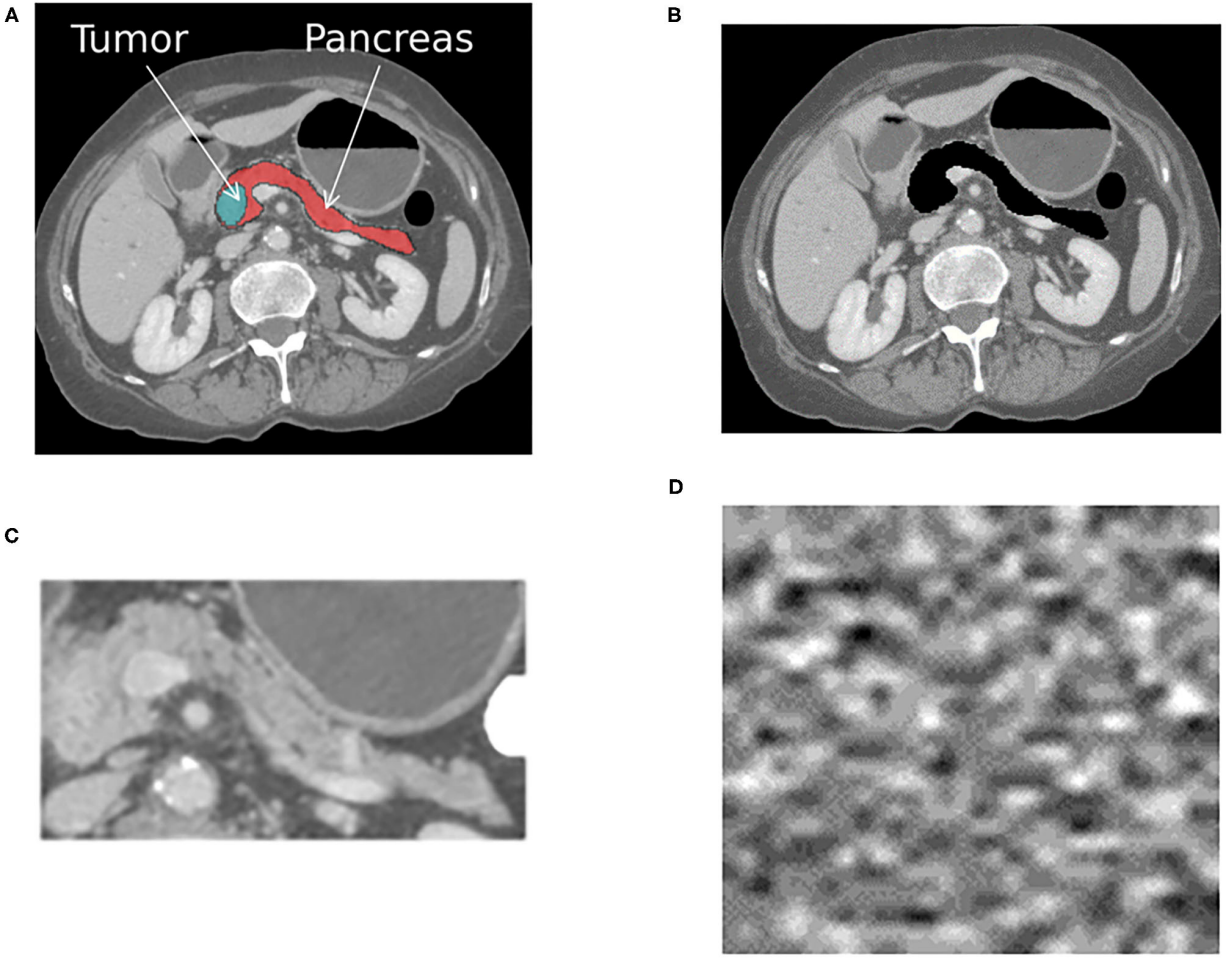
The figure shows an example cross-sectional slice from a single patient with pancreatic cancer that was processed into four different input formats, as shown. A different AI system was trained with each input format, resulting in four different systems that were then tested on each format. **(A)** The original image with the pancreas and tumor present. **(B)** The original image with the pancreas and tumor removed. **(C)** The anatomy surrounding the pancreas is cropped out, and only the pancreas and tumor remain. **(D)** Noise image generated from the patient CT scans. The same processing was applied to all CT scans within the positive for pancreatic cancer and normal pancreas classes.

We propose a series of sanity tests to identify if an AI system may fail during the development phases and before conducting more extensive generalization tests. We also describe how the tests are used with a case study to detect pancreatic cancer from weakly labeled CT scans. The tests are as follows:

- **Train and test with the target-present and absent**. If an AI system is trained to distinguish between normal and abnormal diagnostic features (e.g., organ with cancer shown in Figure 1A), then it should fail when that target is removed from the development test data (e.g., Figure 1B). If the system still works effectively after removing the target from testing data, then that indicates it is confounded. In our case study, this corresponds to removing the pancreas from normal scans and pancreas with tumor from abnormal scans using a segmentation mask, as shown in Figure 1C. We removed the whole pancreas because the pancreatic tumor often distorts the contours of the surrounding anatomy (38).
- **Train and test the system with background patches or noise images**. Background patches consist of non-target regions of the image. Noise images can be generated from the volumetric CT scans in the development and generlization datasets. Both can determine if the different classes can be discriminated based on features unrelated to the target objects (33). If classes are discriminated against with high confidence using the noise image types, then the system is confounded, and it is using features of the image acquisition process to delineate classes. An example noise image generated from the patient CT scans is shown in Figure 1D.
- **Test with different regions of interests (ROIs)**. Training and testing AI systems on precisely outlined segments of images does not reflect real-world usage. Medical centers, private practices, or institutes where AI is deployed may not have the resources or expertise to precisely outline the anatomical area (39). Furthermore, similar to radiologists, AI systems may have to parse through anatomy they have never encountered during training. Therefore, it is desirable to ensure that when systems are trained on a select portion of images, as shown in Figure 1C, they can generalize to the original image shown in Figure 1A.

These sanity tests can be conducted solely using the development dataset, but ideally they would also be used in conjunction with another generalization dataset. They require four input formats, as shown in Figure 1, to be generated from the same development dataset.

### 2.2. Datasets

Institutional review board approval was obtained for this Health Insurance Portability and Accountability Act-compliant retrospective study. The requirement for informed consent was waived. The case study is designed as a binary classification problem with the aim of identifying patients who have pancreatic cancer vs. those who do not from the provided CT scans. We distinguish between the development dataset used for training, tuning and testing, and the generalization test data used to validate the efficacy of the system. The development dataset was processed into four different formats, as shown in Figure 1. The four formats were used to train four different AI systems. The input to each system was a volumetric CT scan that consisted of a normal pancreas or pancreas with tumor. The output from each system was a single classification score that indicated the probability of the patient having pancreatic cancer.

#### 2.2.1. Development Data

The development dataset consisted of patient CT scans collected from two open-access repositories where detailed annotations were available. The normal pancreas CT scans were obtained from The Cancer Imaging Archive Normal (TCIA) Pancreas Dataset with 82 contrast-enhanced abdominal CT scans (40). Seventeen patients from the TCIA dataset were reported to be healthy kidney donors. The remaining patients were selected because they had no major abdominal pathology or pancreatic lesions (40). The abnormal pancreas CT scans were obtained from the Medical Image Segmentation Decathlon (MSD) dataset, consisting of abdominal CT scans from 281 patients. The MSD dataset contains patients who presented with intraductal mucinous neoplasms, pancreatic neuroendocrine tumors, or pancreatic ducal adenocarcinoma (41). They were originally used to predict disease-free survival or assess high-risk intraductal papillary mucinous neoplasms seen on the CT scans (41). We randomly selected 82 cases from the MSD dataset to match the TCIA dataset size to avoid class-imbalance issues. The development data were randomly split into a training (58 normal, 60 cancer), tuning (15 normal, 14 cancer), and held-out test (9 normal, 8 cancer) set. To ensure the number of positive and negative samples were balanced in each split, we used stratified five-fold cross-validation for training. Table 1 shows the patient demographics and scanning parameters provided for each dataset.

**Table 1:** Scan parameters and patient-specific characteristics for development and generalization data.

	**Development Data: Train, Tune, and Test**		
	**TCIA - Pancreas CT**	**Medical Image Segmentation Decathalon (MSD)**	**Generalization data**
Annotated	Yes	Yes	No
CT Vendor	Phillips and Siemens	General Electric	General Electric
CT Model	^**^	LS16 or HD750	HD750
Total # of Patients	82 (27 female/55 male)	281 ^*^	116 (61 female/55 male)
# used to train	58	60	NA
# used to tune	15	14	NA
# used to test	9	8	116 (58 without PC, 58 with PC)
**Dataset Information:**			
Average age (min to max)	46.8 (18 to 76)	^**^	63 (18–90)
Scan start time after contrast administration	~70 s	80–85 s	~40 s
Avg. # of total slices (min/max)	256 (181–466)	95 (37–751)	186 (102–278)
Avg. # of slices consisting of only pancreas (min/max)	85 (45–144)	30 (11–147)	NA
**Scan parameters:**			
Tube potential (kVp)	120	120	70 keV (80/140 kVp)
Slice thickness (mm)	1.5–2.5	2.5	2.5
Pixel dimensions (mm)	0.664 to 0.977	0.606 to 0.977	0.547 to 0.976
Tube current modulation index	^**^	Noise Index: 14 (HD750) / 12.5 (LS16)	NA
Tube current (mA) min to max range	^**^	220–380 mA	260–600
Rotation time (s)	^**^	^****^ 0.7 (HD750) / 0.8 (LS16)	^****^ 0.7 (HD750)
Pitch	^**^	^****^ 0.984 (HD750) / 1.375 (LS16)	^****^0.984 (HD750)
Reconstruction algorithm	^**^	^**^	^***^FBP/ASiR 20%
Reconstruction kernel	^**^	^**^	Standard
Iterative reconstruction strength	^**^	^**^	20%
# of data channels	^**^	^**^	64
Size of a single data channel (mm)	^**^	^**^	0.625
Bowtie filter	^**^	^**^	Large body
CT scan series released or used	Axial portal venous phase	Axial portal venous phase	Axial parenchymal phase

* *A subset of the MSD dataset was randomly selected to train the model*.

** *Not available in accompanied report or DICOM header*.

*** *FBP, Filtered Back Projection; ASiR, Adaptive Statistical Iterative Reconstruction*.

**** *LS16, LightSpeed16; HD750, Discovery High Definition 750*.

#### 2.2.2. Generalization Data: Dual Energy CT (DECT)

The generalization data consists of 116 patients (58 without PC, 58 with PC) who received routine DECT scans between June 2015 to December 2017 (see Table 1). The patients without pancreatic cancer received DECT CT Urography (CTU) exams and were selected based on the statement of a negative or unremarkable pancreas and liver in the radiologist report. Those with cancer were selected if they had undergone a DECT arterial phase CT scan and were histologically confirmed to have pancreatic cancer. All patients were scanned on a 64 slice CT scanner (Discovery CT750 HD, GE Healthcare, Milwaukee, WI, U.S.) with rapid switching DECT following the administration of 150 mL of iodinated contrast (Iohexol 300 mgI/mL, Omnipaque 300, GE Healthcare, Cork, Ireland), at 4.0 mL/s. The scan parameters are displayed in Table 1. With DECT, multiple image types can be generated, such as virtual monochromatic images (VMI) that depict the anatomy and physiology from the viewpoint of a monochromatic x-ray source (42). The VMI scans can be reconstructed at energies ranging from 40 to 140 keV. For this study, all scans were reconstructed at 70 keV because of its use in the clinic. The images were generated using the GSI MD Analysis software available on Advantage Workstation Volume Share 7 (GE Healthcare). Those patients who had a history of surgery and liver abnormalities were excluded from the test set, as were any patients who had metal adjacent to the pancreas or visible artifacts on the scans. This dataset was not used during the training or tuning stages.

### 2.3. AI System - CTNet

**Figure 2:**
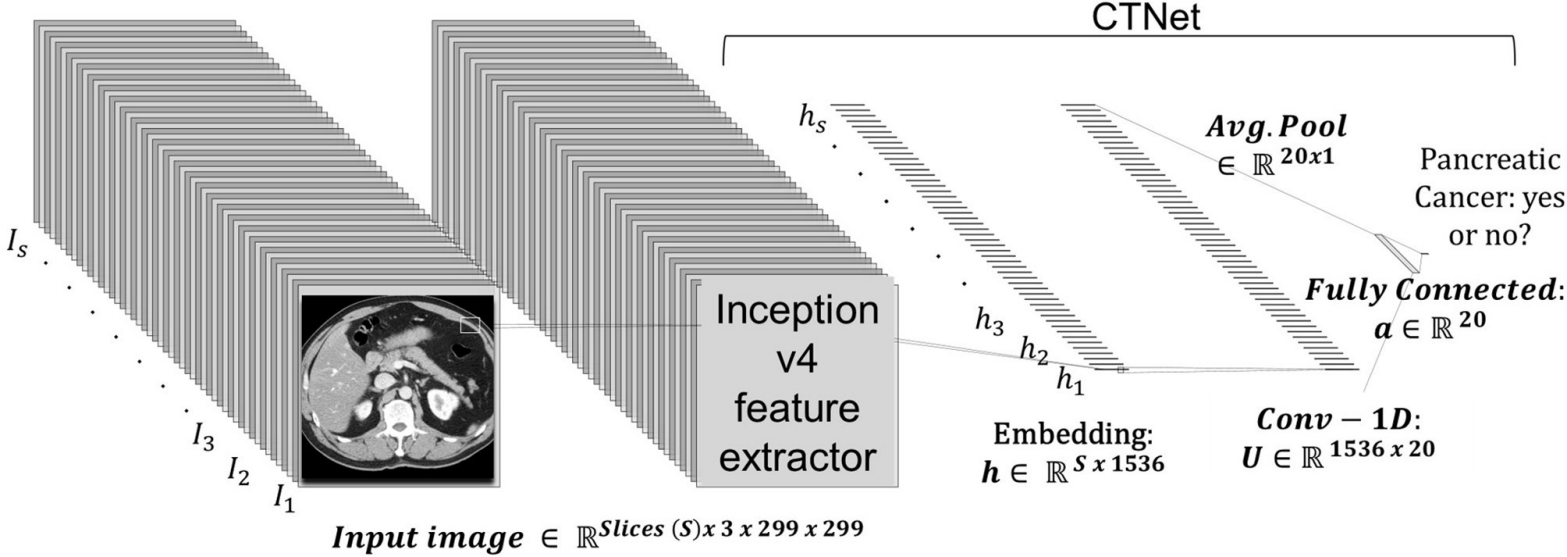
CTNet architecture. CTNet takes as input a volumetric CT scan and outputs a classification prediction. Features are extracted from each slice of the CT scan by the Inception v4 network. The output feature vector is then reduced in dimension with a single convolutional layer, followed by an adaptive average pooling operation applied over the number slices. The resulting vector is fed into a fully connected layer, which has a single output.

The prediction system is dubbed CTNet. It is designed to map a 3D CT scan to a probability estimate that indicates if pancreatic cancer is present or not. CTNet closely resembles systems in literature that use ImageNet pre-trained convolutional neural networks (CNNs) on radiology scans (31, 34, 43–47). The model architecture is shown in Figure 2.

Given a total set of *s* slices in a scan, where each individual slice *t* is a 299 × 299 image, an ImageNet pre-trained Inception v4 CNN was used to extract an embedding $h_{t}\in {\mathbb{R}}^{d}$ from each slice. The embeddings were extracted from the penulmitate layer, which renders a *d* = 1, 536 dimensional feature vector for each image (48). Because Inception v4 is designed to take as input a 299 × 299 × 3 RGB image, we replicated each slice to create faux RGB images. Following others (49), the CNN was not fine-tuned for CT data.

After extracting the embeddings from all scan slices within a CT scan volume, it is then fed into a neural network to make a final prediction, which is given by:


(1) $$\begin{array}{l} P\left(Cancer=1|h_{1},h_{2},\ldots h_{s}\right)\\ \;=\sigma \left(b+\frac{1}{s}w^{T}\sum_{t=1}^{s}\mathrm{ReLU}\left(Uh_{t}+a\right)\right), \end{array}$$

where σ(·) denotes the logistic sigmoid activation function, *b* ∈ ℝ is the output layer bias, **w** ∈ $a\in {\mathbb{R}}^{20}$ is the output layer's weight vector, **U** ∈ ℝ^20 × 1536^ is the hidden layer weight matrix, **a** ∈ ℝ^20^ is the hidden layer bias, and ReLU is the rectified linear unit activation function. In preliminary studies, we found that using 20 hidden units sufficed to achieve strong performance.

The model was trained using the binary cross-entropy loss function with a mini-batch size of 1. The weights were initialized using the Kaiming method. For all systems trained in this study, we used the Adam optimizer with (50) a base learning rate of 1*e*^−4^, *L*_2_ weight decay of 1*e*^−6^, and bias correction terms, β_1_ = 0.9 and β_2_ = 0.999. The learning rate was reduced by a factor of 2 over the course of training when the validation loss had stopped improving. Each system was trained for 100 epochs. Since our training dataset was balanced with positive and negative cases, we did not scale the loss for any particular class's prevalence. During training, no data augmentation techniques were applied. The model was implemented in Python 3.8 with PyTorch 1.6.0 on a computer with a 12 GB NVIDIA Titan V GPU.

### 2.4. Scan Preprocessing

Since the voxel size varied from patient to patient, the CT scans were first resampled to an isotropic resolution of 1.0 × 1.0 × 1.0 mm using SINC interpolation. They were then resized to a height and width of 299 × 299 pixels using bilinear interpolation, which is the original input image size used to train the Inception v4 network. The voxel Hounsfield unit (HU) value was clipped to be between ±300*HU* and normalized to have zero mean and unit variance (i.e., [0, 1]). Normalization was performed by subtracting the mean and dividing by the standard deviation computed from the training dataset. This processing was applied to both the development and generalization datasets.

### 2.5. Noise Image Generation

**Figure 3:**
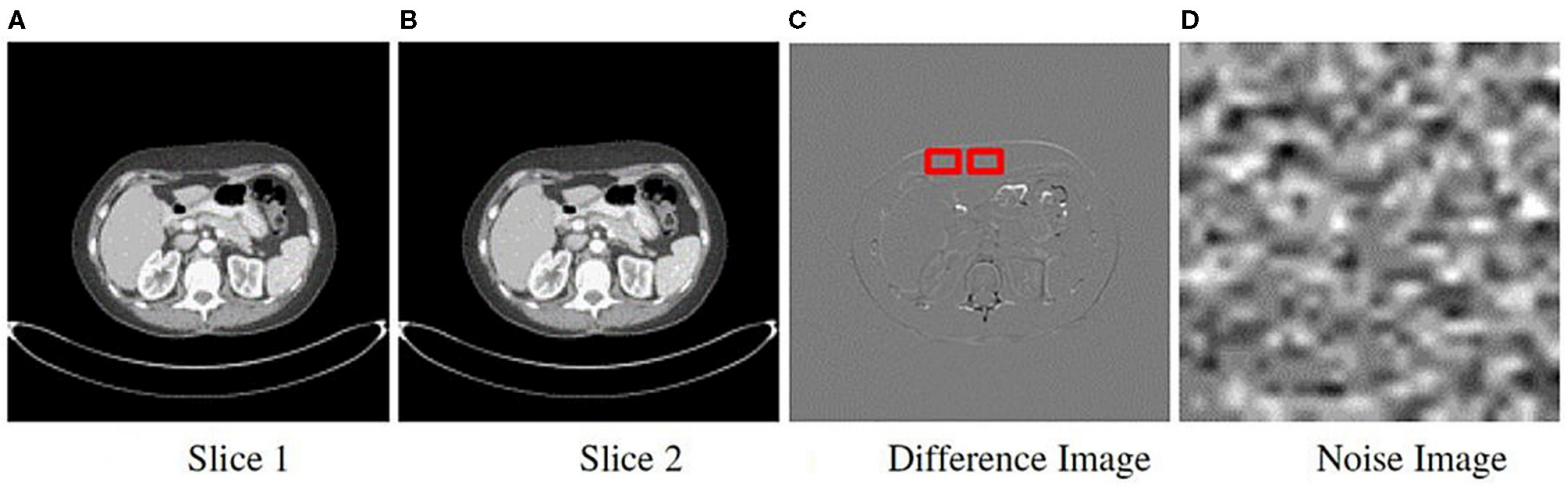
Method to obtain noise maps from sequentially acquired images. Two sequential slice images are subtracted from each other **(A,B)**. **(C)** Difference image resulting from subtraction showing the sliding 30 × 30 pixel window used to extract uniform patches. **(D)** All patches were averaged to generate a single noise image.

**Figure 4:**
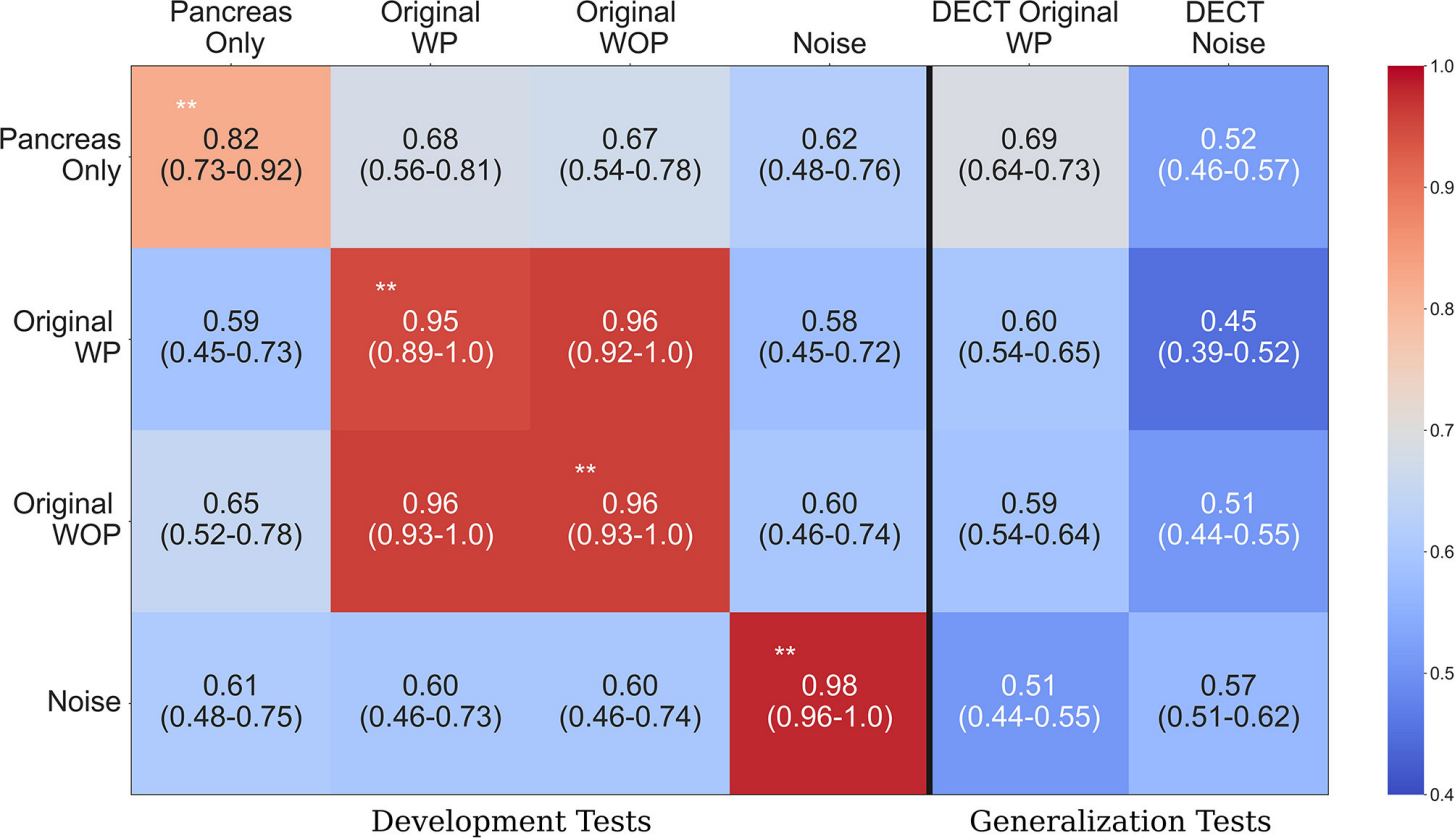
Area under the curve (AUC) heatmap across models for each input format type. Each row indicates the cross-validated mean AUC with 95% confidence intervals for the systems trained with a given input format and evaluated with all other formats from the development dataset (first four columns). The last two columns show the performance of each system on the generalization dataset. The diagonal elements on the development tests correspond to training and testing with the same input format. Red indicates the highest AUC values, while light blue indicates the lowest AUC values. The non-significant difference on the original with pancreas (WP) and without pancreas (WOP) development test sets indicates that spurious correlations drive the performance observed on the self-test sets, instead of features specific to the pancreas or pancreatic cancer. ^**^Development test images processed identically to the data used for training that model. WP, With pancreas; WOP, Without pancreas; DECT, Dual Energy CT.

We derived noise images from the actual scans within each class to determine if the institutional scanning practices or noise characteristics of the imaging systems confound the classification results. As a result, they are composed of unrecognizable or hidden patterns that are a byproduct of the scanner image processing schemes or X-ray detection characteristics. A key attribute of the noise image is that it must be uniform and devoid of any perceptible patterns or structured anatomy. We generated noise images from each patient's CT scan using an approach similar to (51, 52), and as shown in **Figure 4**. For a scan with *s* sequential slices, where each slice *t* is an image $I_{t}\in {\mathbb{R}}^{299\times 299}$, we subtract adjacent slices to produce *s* − 1 difference images *D*_*I*_, where *D*_*I*_ = *I*_*t*_ − *I*_*t*−1_ and 1 ≤ *t* < *s*. The subtraction process eliminates most of the anatomical features seen in the scan. We then apply a Sobel edge enhancing filter to each *D*_*I*_ to identify and remove any remaining anatomical patterns. Then we loop through each *D*_*I*_ to extract non-overlapping patches of size 30 × 30 pixels. The patch size was selected to minimize the impact of the non-uniformity of the CT HU values within the region of interest (e.g., due to streaking or beam hardening artifacts) (52). However, patches of transitional boundary areas (i.e., interface between different tissue types) consisted of discernible patterns that could be spuriously correlated with the class labels. Consequently, to identify and exclude boundary patches, we generated and analyzed each patch's histogram. First-order statistical measures, such as skewness, kurtosis, and standard deviation, and the number of peaks within the histogram were used to identify and exclude boundary patches. Histograms with a skewness value within ±0.1, kurtosis of 3.0 ± 0.5, a standard deviation less than 16, and those with a single peak were included. Published descriptions of the noise image generation method do not provide choices for each of the parameters, so we chose them via visual inspection to eliminate transition areas or edges. The patches that met the criteria were then averaged together to create a single noise image representation of size 30 × 30 for the *D*_*I*_, as shown in Figure 3. Finally, the *s* − 1 noise images for the patient were upsampled using SINC interpolation to a dimension of 299 × 299.

### 2.6. Experiments

To employ the sanity tests, we processed four representations or input formats of the same training, tuning, and held out development test sets. Representative images for a single patient from the cancer positive class are shown in Figure 1. The first format we evaluated were the pancreas-only scans, as shown in Figure 1C. We used the provided annotations to exclude the organs surrounding the pancreas and pancreas with tumor for this format. The cropped portion shown in Figure 1C was resized to an input dimension of 299 × 299 before being fed into CTNet. The second format, referred to as the original with the pancreas (WP) scans and shown in Figure 1A, consisted of the uncropped patient CT scans where the normal or abnormal pancreas was present. For the third format shown in Figure 1B, the pixels that composed the normal or abnormal pancreas were replaced with zeros. These are referred to as the original without a pancreas (WOP) scans. The fourth format, shown in Figure 1D, consisted of the noise images. We trained four systems, one for each input format, and tested each of them with the held-out test sets of the other formats. Since annotations were not available for the generalization test set, we generated two formats: (1) the original uncropped images, which are referred to as DECT original WP, and (2) the noise images, which are referred to as DECT noise scans. We performed stratified five-fold cross-validation with the same division of scans across the four systems. For this study, we consider the baseline against which all results are compared to be the system trained with the pancreas-only scans shown in Figure 1C, as it should be the representation that maximizes the classification performance.

### 2.7. Statistical Analysis

Each system's classification performance was assessed using the area under the receiver operator characteristic curve (AUC). We report the average AUC and corresponding 95% confidence interval (CI) across cross-validation runs. An average AUC score of 1.0 represents perfect classification performance. The average AUC across runs and the corresponding confidence intervals were determined using R (Rstudio version 3.6.2) with the package cvAUC for cross-validated AUC (53). In addition to confidence intervals, statistically significant differences between test runs was confirmed with the DeLong test statistic for AUCs (54). The level of significance was set at *P* ≤ 0.05.

## 3. Results

Table 2 provides an overview of how the sanity tests should be interpreted and implemented in practice. Figure 4 shows the performance of each trained system on the held-out tests and the generalization test set. The diagonal elements for the development tests correspond to training and testing on the same input format (i.e., self-tests), while the others represent AUC scores from training on one format and testing on the other (i.e., non-self tests). We expect a system trained on one format to perform the best on test data processed in an identical manner, which is consistent with the self-test results along the diagonal of Figure 4. For instance, the system trained with the pancreas-only images achieved an AUC of 0.82 (95% CI: 0.73–0.92) on its self-test format. If the system was considered to pass the sanity tests, we would expect it to have the highest AUC across self-test results and the original WP test format. However, instead, it is the lowest among the self-tests. Its performance is significantly lower (*P* < 0.001) than systems trained on the original WP and WOP, 0.95 AUC (95% CI: 0.89–1.0) and 0.97 AUC (95% CI: 0.93–1.0), respectively.

**Table 2:** The proposed sanity tests to assess the reliability of medical AI systems.

**Sanity test**	**Implications of failing the test**	**Does CTNet pass the test?**
**Train and test with and without the target:** The system should achieve an AUC of around 0.5 when tested without the target in test images.	Images contain spurious covariates that can be exploited by the model.	✗
**Train and test using noise images:** The system should achieve an AUC of around 0.5 on test data.	Classification performance cannot be attributed to recognition of the target (i.e., covariates contribute to the learned classification decision rule).	✗
**Test system with different sized ROIs:** The additional or reduced context should not alter the performance.	The system cannot decorrelate features of the target from its co-occurring context [i.e., Contextual Bias (55)].	✗

The second and third rows of Figure 4 show the performance of the systems trained with the original WP and WOP formats. Both systems performed exceptionally well on their self-test sets and each other's test format, but they both saw a drop in performance when they were evaluated with the pancreas-only and noise image test formats (*P* < 0.001). The performance across test formats suggests that the confounding variables within the development data are readily associated with the image-level labels. Several sources of bias may be responsible for the observed results, such as the differences in scan parameters, types of scanners, choice of reconstruction algorithms, and the distribution of contrast within the pancreas.

The noise-only system achieved the highest AUC of 0.98 (95% CI: 0.96, 1.0) among the self-test sets, suggesting that discriminative but unrecognizable features unique to each dataset were used to distinguish each class. The results observed on the development data are confirmed on the generalization tests where each system's performance is significantly lower than on the development data self-tests (*P* < 0.001). For example, the average AUC achieved by the pancreas-only system on the self-test was 0.82 (95% CI: 0.73, 0.92), but its performance was significantly lower on the DECT original WP generalization test format (*P* < 0.001). One reason for the reduced performance on the generalization dataset is the difference in scan parameters with the development datasets. The DECT scans are synthesized images that depict anatomy from the viewpoint of a monochromatic X-ray source. However, within the clinic, AI systems may be tasked with assessing scans acquired on any type of CT system.

## 4. Discussion

Identifying covariates that cause unintended generalization or those that cause machines to fail unexpectedly in deployment remains a challenge across deep learning applications. We described sanity tests that could reveal if covariates drive classification decision-making and tested them with a case study designed to classify pancreatic cancer from CT scans. Failing these sanity tests provides an early indicator of potential biases being responsible for the observed performance and that further in the development process, a system will unintentionally generalize or have much lower performance when deployed. We argue that others should routinely use these tests in publications. For industry, these tests could save time and money. Failing them indicates that the target objects' attributes are not being used by the systems undergoing analytical and clinical validation studies. Hence, as we show, relying only on conventional testing strategies with development data will not provide adequate assurances of generalization. Our sanity tests can be used with development data as long as ROIs are available or a background noise image can be generated. While we focused on binary classification, the sanity tests apply to the multi-class classification and regression problems, with appropriate statistical analysis modifications.

The proposed sanity tests are designed to identify early when an AI system reaches the correct classification for the wrong reasons, but they are not designed to identify the reason for the incorrect decision. As far as what those reasons are, it may not be possible to tease them apart given the limitations of public datasets where private and non-private meta-data are removed. For instance, both NIH (40) and MSD (41) reports did not indicate if iterative reconstruction (IR) was used, the size or number of data channels used to acquire images, and the Bowtie filter. These parameters were also not present in the DICOM meta-data. Modern CT scanners often use varying strengths of IR to suppress image noise, but with the application of IR, images appear smoother, and depending on the strength, the noise texture becomes finer or more coarse (56–58). Another source of bias is the quality of the annotations and accuracy of information released about a public dataset (59). For example, Suman et al. found that parts of the pancreas were absent in the provided segmentation's for the NIH pancreas-CT dataset (59). In addition, we observed a discrepancy between the reported slice thickness from the MSD dataset (41) and existing information in the DICOM meta-data. The report indicates that all scans were acquired using a slice thickness and reconstruction width of 2.5, but the information derived from the DICOM meta-data shows that the slice thickness's for some scans was: 0.70, 1.25, 1.5, 2.0, 2.5, 3.75, 4.0, 5, and 7.5 mm. A common slice thickness could prevent resampling errors (e.g., aliasing) that may arise from down or up-sampling the CT scans. The discrepancy and missing meta-data motivates the need for data-reporting standards and standardized study designs with more rigorous validation procedures.

We did not attempt to use techniques to mitigate the impact of spurious correlations. These include adversarial regularization (19, 60, 61), model ensembling (62, 63), invariant risk minimization (64, 65) and methods that encourage grounding on causal factors instead of spurious correlations (66–69). However, as shown by Shrestha et al. (70), methods that were thought to overcome spurious correlations were behaving as regularizers instead of overcoming the issues that stemmed from the covariates. Our sanity tests could be used with these mitigation methods to measure their true impact, in that we would expect them to only be able to provide significant benefit when the target is present.

There are some limitations to this study. As with most AI studies involving medical image analysis, we trained and tested with a small dataset and did not account for spectral or disease prevalence biases within development or generalization data. Results stemming from small-data may not always transfer to scenarios where larger datasets are used to train systems, but this is in part why sanity tests when using smaller datasets are critical since it is likely easier for spurious correlations to impact them. Also, since the goals of this study were to define sanity tests and illustrate their application, we did not investigate the reasons behind the reduced performance on the generalization data. However, the reduced performance could be a byproduct of the divergent scan parameters and difference in scan type (i.e., SECT vs. DECT and scan phase) between the development and generalization datasets. In general, our sanity tests help reveal when an AI model predicts the right answer for the wrong reasons and will therefore have a large gap between development and external generalization tests. A complementary approach uses visualization methods to understand if a system is not looking at the target to perform its classification.

In conclusion, we demonstrated how our proposed sanity tests could identify spurious confounds early, using development data solely. While the methods are simple, we argue that sanity tests similar to these should be performed wherever possible, especially with smaller datasets, and if no external dataset is available. Otherwise, study results can be very misleading and fail to generalize on other datasets. In safety-critical AI domains, such as healthcare, sanity tests could prevent harm to patients, and they could better prepare novel medical AI systems for regulatory approval. We present a workflow and practical sanity tests that can reliably reveal error-prone systems before influencing real-world decision-making.

## Data Availability Statement

The datasets presented in this article are not readily available because Institutional policy and privacy laws. Requests to access the datasets should be directed to mahmoodu@mskcc.org.

## Author Contributions

UM and CK conceived the study. UM implemented the algorithms and carried out the experiments. UM, CK, and RS wrote the paper. DB, LM, GC, and YE helped gather the data, provided the advice, and reviewed the manuscript. All authors contributed to the article and approved the submitted version.

## Conflict of Interest

CK was employed at Paige, a commercial company, during the preparation of this manuscript. This company played no role in the sponsorship, design, data collection and analysis, decision to publish, or preparation of the manuscript. The remaining authors declare that the research was conducted in the absence of any commercial or financial relationships that could be construed as a potential conflict of interest.

## Publisher's Note

All claims expressed in this article are solely those of the authors and do not necessarily represent those of their affiliated organizations, or those of the publisher, the editors and the reviewers. Any product that may be evaluated in this article, or claim that may be made by its manufacturer, is not guaranteed or endorsed by the publisher.
